# Association of plasma xanthine oxidoreductase activity with blood pressure affected by oxidative stress level: MedCity21 health examination registry

**DOI:** 10.1038/s41598-020-61463-8

**Published:** 2020-03-10

**Authors:** Shio Yoshida, Masafumi Kurajoh, Shinya Fukumoto, Takayo Murase, Takashi Nakamura, Hisako Yoshida, Kazuto Hirata, Masaaki Inaba, Masanori Emoto

**Affiliations:** 1 Department of Metabolism, Endocrinology, and Molecular Medicine, Osaka City University Graduate School of Medicine, Osaka, Japan; 2 Department of Premier Preventive Medicine, Osaka City University Graduate School of Medicine, Osaka, Japan; 3 Mie Research Laboratories, Sanwa Kagaku Kenkyusho Co., Ltd., Inabe, Mie Japan; 4 Department of Medical Statistics, Osaka City University Graduate School of Medicine, Osaka, Japan; 5 Osaka City University, Osaka, Japan

**Keywords:** Cardiology, Medical research

## Abstract

Xanthine oxidoreductase (XOR) inhibitor administration reduces uric acid and reactive oxygen species (ROS) production, and also lowers blood pressure (BP). However, the associations of plasma XOR activity, uric acid level, and oxidative stress levels with BP remain unclear. This cross-sectional study included 156 subjects (68 males, 88 females) registered in the MedCity21 health examination registry without anti-hypertensive or anti-hyperuricemic agent administration. Plasma XOR activity was measured using our highly sensitive novel assay, which is unaffected by uric acid in the sample. BP was also determined, and serum uric acid and derivative of reactive oxygen metabolites (d-ROMs) levels were simultaneously measured. Median plasma XOR activity, serum uric acid, d-ROMs, and mean arterial pressure (MAP) values were 25.7 pmol/h/mL, 5.4 mg/dL, 305 Carr U, and 89.0 mmHg, respectively. Multiple regression analysis showed that plasma XOR activity (β = 0.211, p = 0.019), but not serum uric acid (β = 0.072, p = 0.502), was significantly associated with MAP. In subjects with lower but not higher d-ROMs level, an independent association of plasma XOR activity with MAP was observed (β = 0.428, p = 0.001 and β = 0.019, p = 0.891, respectively; p for interaction = 0.046). XOR may contribute to the pathophysiology of higher BP through ROS but not uric acid production, especially in patients with lower oxidative stress.

## Introduction

Xanthine oxidoreductase (XOR) is a rate-limiting enzyme shown to be involved *in vivo* in production of not only uric acid but also reactive oxygen species (ROS)^1,2^. Accumulating evidence indicates that either uric acid or XOR can cause vascular injury following endocytosis by vascular endothelial cells^3–6^. Although meta-analysis findings have demonstrated that administration of an XOR inhibitor reduces blood pressure (BP)^7,8^, it has yet to be determined whether uric acid or XOR have a major role in regulating BP.

We recently developed a highly sensitive test for human plasma XOR activity^9,10^, which utilizes an assay of stable isotope-labeled [^13^C_2_,^15^N_2_] xanthine with liquid chromatography (LC)/triple quadrupole mass spectrometry (TQMS), thus is unaffected by the original uric acid concentration in the sample. In our previous study, despite a significant and independent association of plasma XOR activity with serum uric acid level^11^, plasma XOR activity but not serum uric acid level was significantly associated with decreased flow-mediated dilatation (FMD) (preliminary findings), suggesting that XOR contributes to the pathophysiology of endothelial dysfunction through ROS production.

ROS have been implicated to have a role in elevated BP through composite mechanisms, including endothelial dysfunction, vascular inflammation, increased reactivity, and structural remodeling^12^. However, only a limited study that examined the association of circulating XOR activity with BP in normotensive subjects has been presented^13^, while no investigation of the associations of plasma XOR activity, as well as oxidative and anti-oxidative stress levels with BP, independent of age, gender, and adiposity, has been reported.

In the present study, we used our novel XOR assay method to examine the associations of XOR activity, uric acid and oxidative stress levels, and anti-oxidative potential with BP in subjects registered in the MedCity21 health examination registry.

## Results

### Clinical characteristics of subjects

The characteristics of the enrolled subjects are shown in Table 1. The median values for mean arterial pressure (MAP), systolic BP (SBP), and diastolic BP (DBP) were 89.0, 119.0, and 73.0 mmHg, respectively, and those for uric acid and plasma XOR activity were 5.4 mg/dL and 25.7 pmol/h/mL, respectively. Furthermore, median values for results of derivative of reactive oxygen metabolites (d-ROMs) and biological antioxidant potential (BAP) testing were 305 Carr U and 2104.5 μmol/L, respectively.

**Table 1:** Clinical characteristics of subjects (n = 156).

Age, years	53.0 (45.0–63.3)
Males, n	68 (43.6)
Smoking habit, n	32 (20.5)
BMI	22.1 (20.3–24.2)
VFA, cm^2^	57.7 (35.1–95.0)
SBP, mmHg	119.0 (109.0–131.3)
DBP, mmHg	73.0 (66.0–82.0)
MAP, mmHg	89.0 (80.7–97.7)
FPG, mg/dL	99.0 (93.8–107.0)
HbA1c, %	5.6 (5.5–5.9)
eGFR, mL/min/1.73 m^2^	77.6 (67.3–88.0)
PRA, ng/mL/h	0.9 (0.6–1.4)
PAC, pg/mL	117.5 (93.2–158.0)
ARR	130.2 (84.6–210.4)
d-ROMs, Carr U	305 (277–340)
BAP, µmol/L	2104.5 (2007.3–2229.4)
Uric acid, mg/dL	5.4 (4.2–6.3)
Plasma XOR activity, pmol/h/mL	25.7 (15.0–50.5)

Data are expressed as the median (interquartile range or %).

Abbreviations: BMI, body mass index; VFA, visceral fat area; SBP, systolic blood pressure; DBP, diastolic blood pressure; MAP, mean arterial pressure; FPG, fasting plasma glucose; HbA1c, glycated hemoglobin; eGFR, estimated glomerular filtration rate; PRA, plasma renin activity; PAC, plasma aldosterone concentration; ARR, aldosterone-to-renin ratio; d-ROMs, derivative of reactive oxygen metabolites; BAP, biological antioxidant potential; XOR, xanthine oxidoreductase

### Positive and independent association of plasma XOR activity, but not serum uric acid, with BP

**Figure 1:**
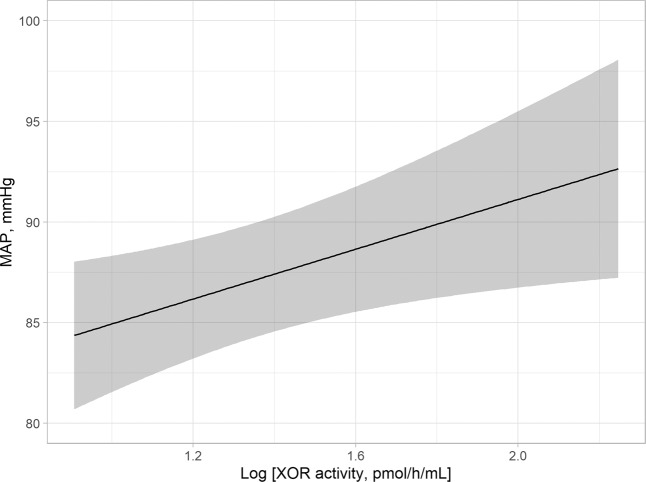
Plasma XOR activity independently associated with MAP in entire cohort. The best fitted line and 95% confidence interval are shown by a solid line and gray band, respectively. MAP was adjusted to the median values for age (53 years), gender (female), VFA (57.7 m^2^), smoking habit (absence), HbA1c (5.6%), eGFR (77.6 mL/min/1.73 m^2^), ARR (130.2), and uric acid level (5.4 mg/dL). Abbreviations: XOR, xanthine oxidoreductase; MAP, mean arterial pressure; VFA, visceral fat area; HbA1c, glycated hemoglobin A1c; eGFR, estimated glomerular filtration rate; ARR, aldosterone-to-renin ratio.

Multivariable linear regression analyses were performed to examine whether plasma XOR activity was independently associated with BP after adjustment for other confounding factors, including age, gender, visceral fat area (VFA), smoking habit, glycated hemoglobin A1c (HbA1c), estimated glomerular filtration rate (eGFR), aldosterone-to-renin ratio (ARR), and uric acid (Table 2). Plasma XOR activity, but not uric acid level, was significantly and positively associated with MAP (β = 0.211, p = 0.019) (Fig. 1), SBP (β = 0.200, p = 0.025), and DBP (β = 0.192, p = 0.038). Furthermore, the regression model was internally validated and the estimated optimism levels were 0.123, 0.112, and 0.137, respectively, indicating no overfitting. Additionally, VFA was significantly and positively associated with MAP, as well as with SBP and DBP, while age was significantly and positively associated with SBP. Gender, smoking habit, HbA1c, eGFR, and ARR each had no significant relationship with MAP, SBP, or DBP.

**Table 2:** Clinical factors possibly affecting BP shown in multivariable linear regression analyses.

	MAP		SBP		DBP	
	β	p	β	p	β	p
Age	0.174	0.074	0.221	0.022	0.120	0.229
Gender (male = 1, female = 0)	−0.026	0.774	−0.146	0.107	0.059	0.526
Visceral fat area	0.296	0.002	0.344	<0.001	0.224	0.021
Smoking habit (present = 1, absent = 0)	0.055	0.456	0.018	0.801	0.074	0.334
HbA1c	−0.003	0.970	0.046	0.583	−0.036	0.674
eGFR	0.169	0.073	0.123	0.185	0.179	0.064
ARR	0.092	0.203	0.065	0.365	0.100	0.181
Uric acid	0.072	0.502	0.005	0.960	0.108	0.323
Log XOR activity	0.211	0.019	0.200	0.025	0.192	0.038
Adjusted R^2^/p	0.244/<0.001	0.266/<0.001	0.207/<0.001			

Values shown represent standardized partial regression coefficient (β values) and level of significance.

R^2^: coefficient of determination.

Abbreviations: BP, blood pressure; MAP, mean arterial pressure; SBP, systolic blood pressure; DBP, diastolic blood pressure; HbA1c, glycated hemoglobin; eGFR, estimated glomerular filtration rate; ARR, aldosterone-to-renin ratio; XOR, xanthine oxidoreductase

### Association of plasma XOR activity with MAP independent of oxidative stress level or anti-oxidative potential

To further examine whether the association of plasma XOR activity with MAP is independent of oxidative stress level or anti-oxidative potential, multivariable linear regression analyses were again performed (Table 3). When the d-ROMs and BAP test results were added as independent variables to the above-mentioned model, plasma XOR activity (β = 0.216, p = 0.018; and β = 0.212, p = 0.020, respectively) retained a significant and positive association with MAP, whereas no significant association with d-ROMs (β = 0.046, p = 0.550) or BAP (β = 0.033, p = 0.651) was noted. The regression model was internally validated, and the estimated optimism levels were 0.139 and 0.140, respectively, indicating no overfitting.

**Table 3:** Impact of oxidative stress level or anti-oxidative potential in addition to plasma XOR activity on MAP shown in multivariable linear regression analyses.

	Model 1		Model 2	
	β	p	β	p
Age	0.167	0.090	0.173	0.076
Gender (male = 1, female = 0)	−0.006	0.954	−0.026	0.777
Visceral fat area	0.302	0.002	0.305	0.002
Smoking habit (present = 1, absent = 0)	0.050	0.506	0.057	0.447
HbA1c	−0.004	0.965	−0.003	0.968
eGFR	0.163	0.087	0.174	0.068
ARR	0.091	0.211	0.093	0.204
Uric acid	0.058	0.596	0.070	0.515
Log XOR activity	0.216	0.018	0.212	0.020
d-ROMs	0.046	0.550		
BAP			0.033	0.651
Adjusted R^2^/P	0.241/<0.001	0.240/<0.001		

Values shown represent standardized partial regression coefficient (β values) and level of significance.

R^2^: coefficient of determination.

Abbreviations: MAP, mean arterial pressure; HbA1c, glycated hemoglobin; eGFR, estimated glomerular filtration rate; ARR, aldosterone-to-renin ratio; XOR, xanthine oxidoreductase; d-ROMs, derivative of reactive oxygen metabolites; BAP, biological antioxidant potential

### Association of plasma XOR activity with MAP stratified by oxidative stress level or anti-oxidative potential

**Figure 2:**
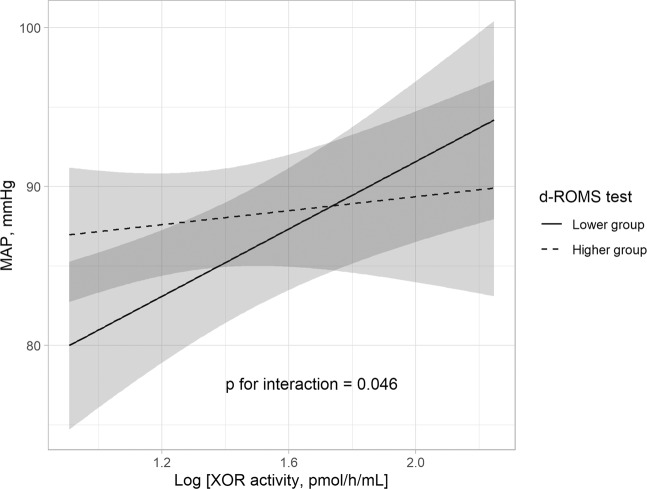
Significant association of plasma XOR activity with MAP in subjects with lower but not higher oxidative stress level. Plasma XOR activity was independently associated with MAP in subjects with a lower d-ROMs level (median <305 Carr U), but not in those with a higher d-ROMs level (median ≥305 Carr U). The best fitted line and 95% confidence interval are shown by a solid line and dark gray band, respectively. MAP was adjusted to the median values for age, gender, VFA, smoking habit, HbA1c, eGFR, ARR, and uric acid level after dividing the subjects based on lower and higher d-ROMs level. Abbreviations: XOR, xanthine oxidoreductase; MAP, mean arterial pressure; d-ROMs, derivative of reactive oxygen metabolites; VFA, visceral fat area; HbA1c, glycated hemoglobin A1c; eGFR, estimated glomerular filtration rate; ARR, aldosterone-to-renin ratio.

We also determined whether the relationship between plasma XOR activity and MAP was modified by oxidative stress level or anti-oxidative potential using interaction analysis (Table 4). The “oxidative stress level * log plasma XOR activity” interaction was significant (p = 0.046), and plasma XOR activity was shown to be significantly and positively associated with MAP in subjects with a lower d-ROMs level (β = 0.428, p < 0.001), whereas no such association was found in those with a higher d-ROMs level (β = 0.045, p = 0.699) (Table 4, Fig. 2), suggesting that a reduced level of oxidative stress has an effect on the relationship between plasma XOR activity and MAP. In addition, plasma XOR activity was significantly and positively associated with MAP in subjects with a lower BAP level (β = 0.293, p = 0.021), but not in those with a higher level (β = 0.046, p = 0.738), though the “anti-oxidative potential * log plasma XOR activity” interaction did not reach a level of statistical significance (p = 0.353). On the other hand, serum uric acid showed no significant association with MAP, even in subjects stratified by oxidative stress level and anti-oxidative potential (Supplementary Table S1). Together, these results suggest that a reduction in plasma XOR activity may have an effect to lower BP, especially in patients with a lower level of oxidative stress.

**Table 4:** Subgroup analysis of association of plasma XOR activity with MAP stratified by oxidative stress level or anti-oxidative potential.

	MAP		P for interaction
	β	p	
Higher d-ROMs level	0.045	0.699	0.046
Lower d-ROMs level	0.428	<0.001	
Higher BAP level	0.046	0.738	0.353
Lower BAP level	0.293	0.021	

β values shown represent standardized partial regression coefficient.

Associations of plasma XOR activity with MAP were adjusted for age, gender, VFA, smoking habit, HbA1c, eGFR, ARR, and uric acid.

Abbreviations: XOR, xanthine oxidoreductase; MAP, mean arterial pressure; HbA1c, glycated hemoglobin; eGFR, estimated glomerular filtration rate; ARR, aldosterone-to-renin ratio

### Association of plasma XOR activity but not serum uric acid with hypertension

Of the 156 subjects, 69 were diagnosed with hypertension. Plasma XOR activity [34.2 (21.8–89.8) vs. 18.6 (11.8–36.1) pmol/h/mL] was significantly (p < 0.001) higher in subjects with as compared to those without hypertension. To examine whether plasma XOR activity was independently associated with hypertension, multivariable logistic regression analyses were performed (Table 5). Plasma XOR activity [odds ratios (OR): 6.554, 95% confidence interval (CI): 1.812–23.706, p = 0.004], but not uric acid level [OR: 1.080, 95% CI: 0.718–1.626, p = 0.712], was significantly and positively associated with hypertension. Age was also found to have a significant association, while VFA only showed a tendency to be associated with hypertension. On the other hand, our findings found no significant association regarding gender, smoking habit, HbA1c, eGFR, or ARR.

**Table 5:** Multivariable logistic regression analysis of factors associated with hypertension.

	OR (95% CI)	p
Age, years	1.049 (1.006–1.094)	0.026
Gender, male = 1, female = 0	0.547 (0.200–1.495)	0.240
Visceral fat area, cm^2^	1.011 (1.000–1.023)	0.059
Smoking habit, present = 1, absent = 0	0.901 (0.337–2.412)	0.836
HbA1c, %	1.242 (0.464–3.319)	0.666
eGFR, mL/min/1.73 m^2^	1.024 (0.988–1.062)	0.187
ARR, ratio	1.001 (0.999–1.004)	0.269
Uric acid, mg/dL	1.080 (0.718–1.626)	0.712
Log XOR activity, pmol/h/mL	6.554 (1.812–23.706)	0.004

Abbreviations: HbA1c, glycated hemoglobin; eGFR, estimated glomerular filtration rate; ARR, aldosterone-to-renin ratio; XOR, xanthine oxidoreductase; OR, odds ratio; CI, confidence interval

## Discussion

The present results demonstrated that plasma XOR activity, but not serum uric acid level, was indepenently and positively associated with BP and hypertension in MedCity21 health examination registry subjects who were not taking anti-hypertensive or anti-hyperuricemic agents (Tables 2 and 5, Fig. 1). In addition, a significant independent association of plasma XOR activity with MAP was found in subjects with a lower but not higher level of d-ROMs (Table 4, Fig. 2). Together, our findings indicate that XOR plays an important role in regulation of BP through ROS, but not uric acid production, especially in patients with a lower level of oxidative stress.

Although a previous study suggested that XOR activity is positively associated with BP in normotensive subjects, the XOR activity assay method used was based on generation of superoxide anion after addition of ferricytochrome c and xanthine^13^. It is important to note that this method was developed to measure XOR activity in rat plasma^14^, while a more consistent and lower plasma level of XOR activity has been shown in humans as compared to rodents^15^. Thus, results of measurements of human plasma XOR activity with that method may not be accurate. In addition, those previous findings did not indicate whether the association between XOR activity and BP was independent of age, gender, or adiposity, such as BMI or VFA, each of which are known to have major effects on BP as well as plasma XOR activity. Thus, the present study is the first to show significant associations of plasma XOR activity with both BP and hypertension, following full adjustments for clinical parameters such as age, gender, and quantitatively determined visceral adiposity by use of a proven accurate method for detection of XOR activity in plasma.

Previously reported meta-analysis findings indicate that XOR inhibitor administration lowers BP^7,8^. In another study, polymorphisms of the XOR gene were found to be related to increased blood pressure and oxidative stress marker levels in patients with hypertension^16^, while experimental studies have shown that XOR contributes to impaired endothelial function by inhibition of nitric oxide-dependent signal transduction through ROS production^17,18^. Furthermore, a recent meta-analysis indicated that administration of allopurinol significantly increases FMD and improved FMD was independent of its uric acid lowering effect^19^. Recently, we found that plasma XOR activity, but not serum uric acid level, had a significant inverse association with FMD (preliminary findings). In the present study as well, plasma XOR activity, but not serum uric acid level, was found to be significantly associated with BP and hypertension, independent of other confounding factors, including VFA and HbA1c, as well as age, gender, smoking habit, eGFR, and ARR (Tables 2 and 5, Fig. 1). Together, these results suggest that XOR contributes to increase BP, at least in part via endothelial dysfunction through ROS production. Importantly, VFA (ρ = 0.513, p < 0.001) and HbA1c (ρ = 0.260, p = 0.001) were positively correlated with plasma XOR activity, which is consistent with previous reports including ours showing that obesity and glycemic control level are positively associated with plasma XOR activity^20,21^. Visceral fat accumulation and diabetes are known to be risk factors for development of hypertension, though the underlying mechanisms have yet to be fully elucidated. The present results suggest that increased XOR activity caused by visceral fat accumulation and diabetes is involved, at least in part, in development of hypertension.

While ROS have been implicated in elevated BP^12^, oxidative stress level was also shown to be increased in subjects with risk factors related to hypertension, such as older age, visceral fat accumulation, diabetes, and renal failure^22^. d-ROMs and BAP testing have been reported to be simple and effective for detecting oxidative stress level and anti-oxidative po tential^23^. Another study showed that d-ROMs test results were positively correlated with those obtained with testing of BAP^24^, considered to be an adaptive response of the body to an increase in ROS production^25^. Consistent with those reports, d-ROMs test results were positively (ρ = 0.159, p = 0.047) correlated with those of BAP in the present study. Of great interest, though the association of plasma XOR activity with BP was not influenced by addition of d-ROMs or BAP test results as a covariate in multivariable regression analyses (Table 3), the association of plasma XOR activity with MAP was significant only in subjects with lower values in those tests as compared to subjects with higher values (Table 4, Fig. 2). A systematic review that included meta-analysis findings^26^ showed that the association of hyperuricemia with development of hypertension was pronounced in younger individuals and females, who were considered to have lower oxidative stress levels. We also found that the association of plasma XOR activity with FMD was prominent in the present participants with lower risk for endothelial dysfunction, such as normotensive and non-diabetic subjects (preliminary findings). Those previous results along with the present indicate that the effect of XOR on BP regulation is diminished by higher oxidative stress conditions, while the contribution of XOR in regulating BP may be more important in subjects with reduced oxidative stress. However, oxidative stress is not regulated by XOR alone, as multiple factors are involved, including nicotinamide adenine dinucleotide phosphate oxidase, nitric oxide synthase, mitochondrial electron transport chain, superoxide dismutase, catalase, and glutathione peroxidase^27^. A future comprehensive study is necessary to clarify the role of XOR-derived oxidative stress in regulation of BP.

Although hyperuricemia is known to predict development of hypertension^26,28^ and XOR inhibitor administration has been reported to lower BP^7,8^, many^29–32^, though not all^33^ studies have found no effect from administration of uricosuric agents such as benzbromarone on BP. Furthermore, administration of the urate precursor inosine was reported to not to raise BP in subjects with Parkinson’s disease^34^ or multiple sclerosis^35^. In addition, mendelian randomization studies showed no association of gene variants mainly expressing the uric acid transporter with BP^36,37^, though some contrasting findings have also been presented^38–40^. Consistent with those reports, plasma uric acid level in the present study was not significantly or independently associated with BP or hypertension (Tables 2 and 5), thus uric acid may not have an important role in the pathophysiology of hypertension in humans. It is also important to note that though previous epidemiological findings have indicated an association of serum uric acid with BP, those findings might have been based on the association of XOR activity with BP, since our previous results demonstrated a significant and independent association of plasma XOR activity with serum uric acid level^11^.

The present findings have several limitations. This was a cross-sectional study, thus even though relationships were explored in predictive terms, the results cannot be interpreted to show causal relationships. In addition, the number of subjects in the entire cohort was relatively small. A large-scale longitudinal study is necessary to clarify the role of XOR in regard to development of hypertension. Nevertheless, our results clearly demonstrate that plasma XOR activity, but not serum uric acid level, is independently associated with BP, especially in patients with a lower level of oxidative stress.

In conclusion, our results showed a positive and independent association of plasma XOR activity, but not serum uric acid, with BP in subjects who were not taking anti-hypertensive or anti-hyperuricemic agents, and that was more prominent in those with lower d-ROMs test results. It is suggested that XOR contributes to the pathophysiology of increased BP through ROS production, but not uric acid production, especially in patients with a lower level of oxidative stress.

## Subjects and Methods

### Study design

The MedCity21 health examination registry was initiated in April 2015 in a comprehensive manner to elucidate the causes of various diseases occurring in adults, including cancer, diabetes mellitus, cardiovascular disease, cerebrovascular disease, mental disorders, dyslipidemia, hypertension, hyperuricemia, obesity, chronic respiratory disease, liver disease, digestive disease, gynecological diseases, and skin disease, for development of advanced diagnostic techniques, as well as treatment and prevention methods for affected patients^11,41–43^. Individuals who underwent comprehensive medical examinations as part of MedCity21 at the Osaka City University Hospital Advanced Medical Center for Preventive Medicine (Osaka, Japan) were registered. The MedCity21 health examination registry protocol has been approved by the Ethics Committee of Osaka City University Graduate School of Medicine (approval No. 2927). Written informed consent was obtained from all subjects and the study was conducted in full accordance with the Declaration of Helsinki. The present study protocol was approved by the Ethics Committee of Osaka City University Graduate School of Medicine (approval No. 3684) and performed with an opt-out option, as explained in instructions posted on the website of the hospital.

### Participants

Referring to the MedCity21 health examination registry of individuals examined between June 2015 and May 2017, the final 200 sequential subjects who participated in advanced comprehensive medical examinations designed to check the status of lifestyle-related diseases, such as hypertension, diabetes, dyslipidemia, visceral obesity, hyperuricemia, atherosclerosis, and cerebrovascular disease, were selected. For the present analysis, those being treated with an antihypertensive (n = 37), XOR inhibitor (n = 4), or uricosuric (n = 1) agent, or with missing data (n = 2) were excluded. As a result, 156 participants (68 males, 88 females) were enrolled as subjects in the present cross-sectional study.

### Physical and laboratory measurements

Information regarding height, body weight, smoking habit, present and past illness, and use of medication for each subject was obtained. SBP and DBP were determined using a fully automated oscillometric upper-arm blood pressure monitor (TM-2656; A&D, Tokyo, Japan), the accuracy of which has been validated^44^. The measurements were done after the subject had rested for at least 15 minutes in a seated position. MAP was calculated as follows: MAP = DBP + 1/3 [SBP – DBP]. Hypertension was defined as SBP ≥ 130 and/or DBP ≥ 80 m mHg^45^. Blood was drawn after an overnight fast, then biochemical parameters were analyzed using a standard laboratory method and remaining blood samples were stored at -80 °C. HbA1c levels were expressed as National Glycohemoglobin Standardization Program equivalent values (%) using the conversion formula established by the Japan Diabetes Society^46^. eGFR was calculated using an equation designed for Japanese subjects, as previously described^47^. Measurements of plasma renin activity (PRA) and plasma aldosterone concentration (PAC) were performed with a radioimmunoassay kit (SRL Inc., Tokyo, Japan). ARR was calculated as follows: ARR = PAC (expressed in pg/mL)/PRA (expressed in ng/mL/h)^48^.

### Plasma XOR activity

Freshly frozen samples were maintained at −80 °C until the time of the assay, then used to determine plasma XOR activity with a recently established method for assays of stable isotope-labeled [^13^C_2_,^15^N_2_] xanthine with LC/TQMS, as we have previously described^9–11^. Briefly, 100-µL aliquots of plasma were purified using a Sephadex G25 column, then mixed with Tris buffer (pH 8.5) containing [^13^C_2_,^15^N_2_] xanthine as a substrate, nicotinamide adenine dinucleotide^+^, and [^13^C_3_,^15^N_3_] uric acid as the internal standard, with the mixtures then incubated at 37 °C for 90 minutes. Subsequently, they were combined with methanol (500 µL) and centrifuged at 2000 × *g* for 15 minutes at 4 °C. Supernatants were collected and transferred to new tubes, then dried using a centrifugal evaporator. The residues were reconstituted in 150 μL of distilled water and filtered through an ultrafiltration membrane, after which measurements were performed using LC/TQMS. Calibration standard samples were examined for the amount of [^13^C_2_,^15^N_2_] uric acid produced, which was calculated by use of a calibration curve, with XOR activity expressed based on that amount (pmol/h/mL).

### Oxidative stress level and anti-oxidative potential

Oxidative stress level and anti-oxidative potential in serum were evaluated using d-ROMs and BAP testing, respectively, with a free radical elective evaluator system (FREE: Diacron International s.r.l., Grosseto, Italy) that included a spectrophotometric device reader, as previously described^23,24^. The d-ROMs test, with results expressed as Carr units, measures the oxidant ability of a serum sample towards N,N-diethyl-para-phenylendiamine used as an indicator (chromogen). The BAP test, with results expressed as μmol/L, measures the blood concentration of antioxidants as agents that can reduce iron from a ferric (Fe^3+^) to ferrous (Fe^2+^) form.

### Measurement of visceral fat area

Using an abdominal computed tomography device (Supria Grande, Hitachi, Ltd., Tokyo, Japan), we acquired a single 5-mm slice at the level of the umbilicus. VFA values were calculated using the fatPointer software package, ver. 2 (Hitachi, Ltd., Tokyo, Japan), as previously described^11^.

### Statistical analysis

Data are expressed as number (%) or median (interquartile range). Spearman’s correlation coefficient was used to determine correlations between variables. Mann-Whitney’s U test was used to compare variables between groups. Data distribution was evaluated using the Shapiro-Wilk test. Since the distribution of plasma XOR activity was skewed, plasma XOR activity was logarithmically transformed before performing multivariable linear regression and logistic regression analyses. Results of those analyses were then used to determine whether plasma XOR activity was independently associated with BP or presence of hypertension, after adjustments for various clinical parameters. To assess the effects of oxidant and anti-oxidant levels on the relationship between plasma XOR activity and MAP, we incorporated a 2-factor interaction term [(higher or lower d-ROMs test result based on median value* log plasma XOR activity) or (higher or lower BAP test result based on median value* log plasma XOR activity)] in a multivariable linear regression analysis model. The reliability of the multivariable linear regression model was internally validated using the bootstrap method. One hundred fifty sets of bootstrap samples were generated by resampling the original data, then the level of optimism was estimated to determine the degree of overfitting. The R software package, ver. 3.2.2 (R Foundation for Statistical Computing, Vienna, Austria) and Statistical Package for the Social Sciences software, ver. 22.0 (PASW Statistics) were used for data analysis. All reported p values are 2-tailed and a p value of < 0.05 was considered significant for all analyses.

## Supplementary information

Supplementary information.
