# Supplementary material for: Association of plasma xanthine oxidoreductase activity with blood pressure affected by oxidative stress level: MedCity21 health examination registry
Source: Sci Rep. 2020 Mar 10;10:4437. doi: 10.1038/s41598-020-61463-8 (PMC7064483; doi:10.1038/s41598-020-61463-8)
Supplement: Supplementary file 1 — Supplementary information. [file 41598_2020_61463_MOESM1_ESM.docx]

**Association of plasma xanthine oxidoreductase activity with blood pressure affected by** **oxidative stress level: MedCity21 health examination registry**

Shio Yoshida^1^, Masafumi Kurajoh^1^*, Shinya Fukumoto^2^, Takayo Murase^3^, Takashi Nakamura^3^, Hisako Yoshida^4^, Kazuto Hirata^5^, Masaaki Inaba^1^, and Masanori Emoto^1^

^1^Department of Metabolism, Endocrinology, and Molecular Medicine, Osaka City University Graduate School of Medicine, Osaka, Japan

^2^Department of Premier Preventive Medicine, Osaka City University Graduate School of Medicine, Osaka, Japan

^3^Mie Research Laboratories, Sanwa Kagaku Kenkyusho Co., Ltd., Inabe, Mie, Japan

^4^Department of Medical Statistics, Osaka City University Graduate School of Medicine

^5^Osaka City University

***Corresponding author:**

Masafumi Kurajoh, MD, PhD

Department of Metabolism, Endocrinology, and Molecular Medicine

Osaka City University Graduate School of Medicine

1-4-3, Asahi-machi, Abeno-ku, Osaka 545-8585, Japan

Tel: +81-6-6645-3806, Fax: +81-6-6645-3808

Email: m1155129@med.osaka-cu.ac.jp

**Supplementary Table S1. Subgroup analysis of association of serum uric acid with MAP stratified by oxidative stress level or anti-oxidative potential**

|  | MAP | | P for interaction |
| --- | --- | --- | --- |
|  | β | p |  |
| Higher d-ROMs level | 0.050 | 0.682 | 0.511 |
| Lower d-ROMs level | 0.023 | 0.889 |  |
| Higher BAP level | 0.064 | 0.691 | 0.962 |
| Lower BAP level | 0.072 | 0.630 |  |

β values shown represent standardized partial regression coefficient.

Associations of plasma XOR activity with MAP was adjusted for age, gender, VFA, smoking habit, HbA1c, eGFR, ARR, and uric acid.

Abbreviations: XOR, xanthine oxidoreductase; MAP, mean arterial pressure; HbA1c, glycated hemoglobin; eGFR, estimated glomerular filtration rate; ARR, aldosterone-to-renin ratio
